# Supplementary material for: Diegoaelurus, a new machaeroidine (Oxyaenidae) from the Santiago Formation (late Uintan) of southern California and the relationships of Machaeroidinae, the oldest group of sabertooth mammals
Source: PeerJ. 2022 Mar 15;10:e13032. doi: 10.7717/peerj.13032 (PMC8932314; doi:10.7717/peerj.13032)
Supplement: Supplemental Information 1 [file peerj-10-13032-s001.docx]

**Part A. Character List**

1. P2 orientation

(0) oblique

(1) longitudinal

2. P3 protocone

(0) absent

(1) present

3. Transverse projection of P4 protocone

(0) close to paracone

(1) widely separated from paracone

4. M1 paracone and metacone fusion

(0) connate but with separation at apices

(1) nearly completely fused with minimal separation

5. M1 metastylar blade orientation

(0) oblique with a distinct inflection between the metastyle and metacone

(1) more longitudinal with a weak inflection

6. M1 protocone size

(0) 1/3 of crown width

(1) very reduced, 1/4 of crown width

7. M2 protocone size

(0) well-developed

(1) reduced to a cingular cusp

8. p1

(0) present, single-rooted

(1) present, double-rooted

(2) absent

9. p2 roots

(0) double-rooted

(1) single-rooted

10. p3 size

(0) large, >75% the length of m1

(1) reduced, <75% the length of m1

11. p3 paraconid

(0) absent

(1) present

12. p4 protoconid orientation

(0) vertical, cusp symmetrical

(1) somewhat reclined posteriorly, distal carina more vertical than mesial

13. p4 paraconid height

(0) lower than talonid

(1) subequal to talonid

14. p4 talonid width

(0) equivalent to trigonid

(1) broader than trigonid

15. m1-2 trigonid height

(0) protoconid and paraconid tall relative to length

(1) protoconid and paraconid relatively lower and more elongate

16. m2 size

(0) larger than m1, m2 trigonid length no larger than subequal to entire length of m1

(1) much larger than m1, m2 paraconid length subequal to entire length of m1

17. m1-2 metaconids

(0) present

(1) absent

18. m1 entocristid

(0) present, may be weak

(1) absent

19. m1 talonid length

(0) approximately one third the length of the crown

(1) approximately one quarter the length of the crown

20. m2 talonid development

(0) small and at least bicuspid

(1) rudimentary and unicuspid

21. posterior extent of mandibular flange

(0) extends to below p1-2

(1) extends to below p3

22. shape of inferior margin of mandibular flange

(0) triangular

(1) rounded

23. coronoid process height

(0) taller than m2

(1) lower than m2

24. mandibular condyle position

(0) even with tooth row

(1) below tooth row

**Part B. List of Materials Used to Construct the Character Taxon Matrix**

**Institutional abbreviations**—CM, Carnegie Museum of Natural History, Pittsburgh, USA; SDSNH, San Diego Natural History Museum, San Diego, USA; UM, Museum of Paleontology, University of Michigan, Ann Arbor, Michigan, USA; USNM, Department of Paleobiology, United States National Museum of Natural History, Smithsonian Institution, Washington D.C., USA; YPM-PU, Princeton University collection, Yale Peabody Museum, Yale University, New Haven, Connecticut, USA.

*Prototomus phobos*: UM 68075, 74134, YPM-PU 13019; Gingerich and Deutsch (1989)

*Dipsalidictis krausei*: UM 69331; Gunnell and Gingerich (1991)

*Machaeroides simpsoni*: CM 36397, 45115; Dawson et al. (1986)

*Machaeroides eothen*: USNM 17059, 361372; Matthew (1909); Gazin (1946)

*Apataelurus kayi*: CM 11920; Scott (1938)

*Apataelurus pishigouensis*: Tong and Lei (1986); Zack (2019)

*Diegoaelurus vanvalkenburghae*: SDSNH 38343

**Part C. Character Taxon Matrix**

|  |  |  |  |  |  |  |  |  |  | 1 |  | 1 | 1 | 1 | 1 | 1 | 1 | 1 | 1 | 1 | 2 |  | 2 | 2 | 2 | 2 |
| --- | --- | --- | --- | --- | --- | --- | --- | --- | --- | --- | --- | --- | --- | --- | --- | --- | --- | --- | --- | --- | --- | --- | --- | --- | --- | --- |
|  | 1 | 2 | 3 | 4 | 5 | 6 | 7 | 8 | 9 | 0 |  | 1 | 2 | 3 | 4 | 5 | 6 | 7 | 8 | 9 | 0 |  | 1 | 2 | 3 | 4 |
| *Prototomus phobos* | ? | 0 | 0 | 0 | 0 | 0 | 0 | 0 | 0 | 0 |  | 0 | 0 | 1 | 0 | 0 | 0 | 0 | 0 | 0 | 0 |  | - | - | 0 | 0 |
| *Dipsalidictis krausei* | 0 | 0 | 1 | 0 | 0 | 0 | 0 | 0 | 0 | 1 |  | 0 | 1 | 0 | 0 | 0 | 0 | 0 | 0 | 0 | 0 |  | - | - | 0 | 0 |
| *Machaeroides simpsoni* | 0 | 0 | 0 | 0 | 0 | 0 | 0 | 1 | 0 | ? |  | ? | 1 | 0 | 0 | ? | 0 | 0 | ? | ? | ? |  | 0 | 0 | ? | ? |
| *Machaeroides eothen* | 1 | 1 | 1 | 1 | 1 | 1 | 1 | 1 | 0 | 0 |  | 0 | 0 | 0 | 0 | 0 | 0 | 0 | 0 | 0 | 0 |  | 0 | 0 | 0 | 0 |
| *Apataelurus kayi* | ? | ? | ? | ? | ? | ? | ? | 0 | 0 | 1 |  | 0 | 1 | 1 | 1 | 1 | 1 | 1 | 1 | 1 | 1 |  | 1 | ? | 1 | 1 |
| *Apataelurus pishigouensis* | ? | ? | ? | ? | ? | ? | ? | ? | ? | ? |  | ? | 1 | 1 | 1 | 0 | ? | 1 | ? | 1 | ? |  | 1 | ? | ? | ? |
| *Diegoaelurus vanvalkenburghae* | ? | ? | ? | ? | ? | ? | ? | 2 | 1 | 1 |  | 1 | ? | ? | ? | 1 | 0 | 1 | 1 | 0 | 0 |  | 0 | 1 | 0 | ? |

REFERENCES

Dawson, M. R., R. K. Stucky, L. Krishtalka, and C. C. Black. 1986. *Machaeroides simpsoni*, new species, oldest known sabertooth creodont (Mammalia), of the Lost Cabin Eocene. Contributions to Geology, University of Wyoming, Special Paper 3:177-182.

Gazin, C. L. 1946. *Machaeroides eothen* Matthew, the saber-tooth creodont of the Bridger Eocene. Proceedings of the United States National Museum 96:335-347.

Gingerich, P. D., and H. A. Deutsch. 1989. Systematics and evolution of early Eocene Hyaenodontidae (Mammalia, Creodonta) in the Clarks Fork Basin, Wyoming. Contributions from the Museum of Paleontology, The University of Michigan 27:327-391.

Gunnell, G. F., and P. D. Gingerich. 1991. Systematics and evolution of late Paleocene and early Eocene Oxyaenidae (Mammalia, Creodonta) in the Clarks Fork Basin, Wyoming. Contributions from the Museum of Paleontology, The University of Michigan 28:141-180.

Matthew, W. D. 1909. The Carnivora and Insectivora of the Bridger Basin, Middle Eocene. Memoirs of the American Museum of Natural History 9:291-567.

Scott, W. B. 1938. A problematical cat-like mandible from the Uinta Eocene, *Apatælurus kayi*, Scott. Annals of Carnegie Museum 27:113-120.

Tong, Y., and Y. Lei. 1986. Fossil creodonts and carnivores (Mammalia) from the Hetaoyuan Eocene of Henan. Vertebrata PalAsiatica 24:210-221.

Zack, S. P. 2019. The first North American *Propterodon* (Hyaenodonta: Hyaenodontidae), a new species from the late Uintan of Utah. PeerJ 7:e8136.
